# The Centenary of the Royal Medical and Chirurgical Society

**Published:** 1905-05-27

**Authors:** 


### The Centenary of the Royal Medical and Chirurgical Society.

There are few medical societies in the United Kingdom which are able to boast that they have passed their hundredth year. It is fitting, therefore, that the centenary of the premier Medical Society in London should be celebrated with something of pomp and circumstance. The social aspect of the medical profession, apart from the scientific side, owes much to John Hunter. He seems to have had as great a genius for founding and carrying to a successful issue various medical societies as he had for propounding new theories, and the sudden loss of his personal influence fell heavily upon them. Hunter founded, or had helped to found, among other societies, the Lyceum Medicum Londinense, which always met in his museum at the back of his house in Leicester Square; and the disruption of this society at his death, coupled with the unhappy state of the Medical Society which had groaned under the tyranny of one president for twenty-two years, gave an opening for the foundation of a new society. This was originated at the Freemasons' Tavern on Wednesday, May 22, 1805. This society was called from the first the Medical and Chirurgical Society of London. It was organised on broad lines and was purposely made somewhat expensive, for the entrance fee was fixed at six guineas and the annual subscription at three guineas. It is remarkable that a society, which became noted for the extreme formality of its business, was established to afford an opportunity for an easy and agreeable exchange of practical knowledge where "written communications shall not be subject to discussion." From the first year of its existence the Society began to collect books, and by a steady progress it has got together the magnificent medical library of which it is now so justly proud. Like all other societies it has had varying fortunes. Its collectors have run away with the subscriptions, it has moved its quarters time and again, it has been torn by internal dissensions, and it has had a curiously fluctuating attendance at its meetings. Yet from the beginning it has had the wisdom to move with the times, and by Royal favour it gained a Charter, and has always availed itself of the prestige of Royal patronage. Speaking with Royal tact at the centenary banquet held at the Hotel Cecil on Monday last H.R.H. the Prince of Wales said: "I consider it a great honour to have been enrolled among the honorary fellows, and to be identified with a Society with which my family has been connected ever since its incorporation by Royal Charter in 1834." The Council of the Society have devoted much thought to the celebration of the present week, and the success with which the various events have been carried out proves the thoroughness with which all the details have been considered. Centenary day began at five o'clock on Monday, when the president, Sir Richard Douglas Powell, received the honorary fellows, and afterwards delivered a short address of congratulation. A banquet followed at which more than 400 fellows dined. On Tuesday Dr. Henry Head, the Marshall Hall prizeman, delivered an address, and there was afterwards an informal reception by the president and Council. The celebration was concluded on Wednesday by a conversazione in the Natural History Museum, the guests being received by the president and Lady Powell. A capable committee under the chairmanship of Dr. J. F. Payne had brought together an exhibition of books, instruments and other objects of interest to illustrate the condition of medicine at the time the Society was established. The exhibition included memorials of various kinds relating to the presidents of the Society, and a series of medals struck to commemorate or to honour fellows. There is thus every evidence that the Royal Medical and Chirurgical Society is in no way effete by lapse of years. It has acquired excellent premises in a central position, and recent events have shown that in the immediate future it may become an integral and perhaps dominating factor in an academy or royal society of medicine.

